# Supplementary material for: The continued influence of AI-generated deepfake videos despite transparency warnings
Source: Commun Psychol. 2026 Jan 2;4:13. doi: 10.1038/s44271-025-00381-9 (PMC12848074; doi:10.1038/s44271-025-00381-9)
Supplement: Supplementary file 2 — Supplementary Information [file 44271_2025_381_MOESM2_ESM.pdf]

Supplementary Information for

The Continued Influence of AI-Generated Deepfake Videos Despite Transparency Warnings

Simon Clark and Stephan Lewandowsky

School of Psychological Science, University of Bristol, UK

Supplementary Methods

Video Transcripts

Experiments 1 and 2

This one-minute video appears to be a clip from a longer video, starting and stopping abruptly. It features John Carter, a fictional local politician, sitting at a table in a bar. He is wearing a suit and open shirt, and holding a glass of wine. He is facing the camera, and a half empty pint of beer on the table suggests he is speaking to someone opposite, although no one else is visible in the video. He speaks quietly but can be heard fairly clearly, in spite of the background noise of a bar. At one point he stops speaking as someone passes the table, suggesting he does not wish to be overheard. The video looks as if it could have been secretly filmed by the person opposite; the angle and lighting are not perfect but John Carter's face is clearly visible.

"... So yeah, obviously this is between you and me, OK, I haven't told anyone else this, not even the wife. ... But yeah, I did it ... yeah I took the money. ... But to be fair, right, I'm pretty sure everyone was doing it, or at least I definitely wasn't the only one. ... Because I mean, what's the point, really, in having ... [PAUSES, as someone passes the table carrying a pint of beer] *... What's the point in having a job like mine, right, if you can't take the odd bribe, OK?* [LAUGHS] *... I mean ... but anyway, ... the most important thing is I'm pretty sure they can't prove it. I mean, it was all in cash, right, and if the police had any real evidence they'd have used it by now, right? ... And they certainly won't find the money, that is safely tucked away in a safety deposit box in Jersey ...* [LAUGHS] *until... well, until this all blows over."*

Experiment 3

This two-minute video features Amelia Palmer, a fictional 27-year-old social media influencer, sitting in a picture-perfect cottage kitchen. She is wearing a simple dress, silver pendant and make-up. Behind her on the kitchen counter is a light box displaying the message "LOVE VEGAN LIFE" to her right, with fresh fruit and vegetables carefully arranged on a vintage wooden chopping board to her left. She looks straight into the camera with a serious expression.

"Hello everyone. I wanted to come here today and talk to you about something that I think I need to address. Umm… obviously this is quite serious and I've upset quite a few people. And so I think it is time that I put the rumours and the whispers to bed, and tell you really what happened. … I am a vegan, and I've always been a vegan, and I've always stood for what I believe in. However, a few weeks ago, at a press event, somebody saw me in a restaurant, eating a burger. … I know that this might sound silly to some people, but to my followers, and to me, this is a really big deal. And I've let you all down. … I wanted to tell you a little bit more about where I was, where my headspace was at, in that moment. … I was in a bad place and I'd come to a point in my life where I was confused about what I was really truly standing for. … But seeing how much I've let you down and disappointed you, how many people have got in touch to say that, the way that they see me and what I do now is, just, false and wrong and a lie, it's really hurt. … And I can only imagine how much that's hurt you. … So all I can say, really, is I'm sorry. I apologise from deep within me, and I want you to know that I've learnt. And if anything, this experience, that day, the way that you have come to me to tell me how it's made you feel, that's made me even more passionate about being a vegan. … We should stand together for what we believe in. … Thank you."
