# Supplementary material for: The continued influence of AI-generated deepfake videos despite transparency warnings
Source: Commun Psychol. 2026 Jan 2;4:13. doi: 10.1038/s44271-025-00381-9 (PMC12848074; doi:10.1038/s44271-025-00381-9)
Supplement: Supplementary file 3 — Reporting Summary [file 44271_2025_381_MOESM3_ESM.pdf]

nature portfolio

Corresponding author(s): Simon Clark
simon.clark@bristol.ac.uk

Last updated by author(s): Aug 29, 2025

Reporting Summary

Nature Portfolio wishes to improve the reproducibility of the work that we publish. This form provides structure for consistency and transparency in reporting. For further information on Nature Portfolio policies, see our Editorial Policies and the Editorial Policy Checklist.

Statistics

For all statistical analyses, confirm that the following items are present in the figure legend, table legend, main text, or Methods section.

n/a	Confirmed	
☐	☒	The exact sample size (*n*) for each experimental group/condition, given as a discrete number and unit of measurement
☐	☒	A statement on whether measurements were taken from distinct samples or whether the same sample was measured repeatedly
☐	☒	The statistical test(s) used AND whether they are one- or two-sided<br>*Only common tests should be described solely by name; describe more complex techniques in the Methods section.*
☒	☐	A description of all covariates tested
☐	☒	A description of any assumptions or corrections, such as tests of normality and adjustment for multiple comparisons
☐	☒	A full description of the statistical parameters including central tendency (e.g. means) or other basic estimates (e.g. regression coefficient) AND variation (e.g. standard deviation) or associated estimates of uncertainty (e.g. confidence intervals)
☐	☒	For null hypothesis testing, the test statistic (e.g. *F*, *t*, *r*) with confidence intervals, effect sizes, degrees of freedom and *P* value noted<br>*Give P values as exact values whenever suitable.*
☒	☐	For Bayesian analysis, information on the choice of priors and Markov chain Monte Carlo settings
☒	☐	For hierarchical and complex designs, identification of the appropriate level for tests and full reporting of outcomes
☐	☒	Estimates of effect sizes (e.g. Cohen's *d*, Pearson's *r*), indicating how they were calculated

Our web collection on statistics for biologists contains articles on many of the points above.

Software and code

Policy information about availability of computer code

Data collection	Experiments were administered online using the Qualtrics survey platform. Raw data were exported to Apache OpenOffice (v4.1) for initial storage and cleaning prior to analysis.
Data analysis	All analyses were conducted using JASP (v0.13).

For manuscripts utilizing custom algorithms or software that are central to the research but not yet described in published literature, software must be made available to editors and reviewers. We strongly encourage code deposition in a community repository (e.g. GitHub). See the Nature Portfolio guidelines for submitting code & software for further information.

Data

Policy information about availability of data

All manuscripts must include a data availability statement. This statement should provide the following information, where applicable:

- Accession codes, unique identifiers, or web links for publicly available datasets
- A description of any restrictions on data availability
- For clinical datasets or third party data, please ensure that the statement adheres to our policy

Preregistrations and full reproduction data are available at osf.io/sjw9h. No custom code was used; all analyses were conducted using standard functionality in JASP (v0.13). This is an Open Access article distributed under the terms of the Creative Commons Attribution License (https://creativecommons.org/licenses/by/4.0/), which permits unrestricted reuse, distribution, and reproduction in any medium, provided the original work is properly cited.

Research involving human participants, their data, or biological material

Policy information about studies with human participants or human data. See also policy information about sex, gender (identity/presentation), and sexual orientation and race, ethnicity and racism.

Reporting on sex and gender	Demographic data, including self-reported gender, were collected for descriptive purposes only and not included in our analyses. No sex or gender based analyses were conducted because our research questions and hypotheses concerned general cognitive and social processes, and experiments were not designed or powered to test sex or gender differences. Our findings are expected to generalize across adult participants, and are not specific to one sex or gender. In accordance with university ethics guidance, demographic variables are not included in the openly shared dataset to protect participant anonymity.
Reporting on race, ethnicity, or other socially relevant groupings	Data on race, ethnicity, or other socially relevant groupings were not collected, because our experiments were designed to test general cognitive and social processes. Our findings are expected to generalize across adult participants.
Population characteristics	See 'Behavioural & social sciences study design' section.
Recruitment	In Experiments 1 and 2, participants were recruited from the United States via the CloudResearch MTurk Toolkit. Experiment 1 participants were paid $1.25 to complete an eight-minute questionnaire, and Experiment 2 participants were paid $1.00 to complete a six-minute questionnaire. In Experiment 3, participants were recruited from the United Kingdom via Prolific, and paid £1.00 to complete a six-minute questionnaire. Participants were self-selected users of these online platforms, and as such may not be fully representative of the wider US or UK populations. However, there is no reason to expect that this would systematically bias the experimental effects of interest, which concern general cognitive and social processes rather than culturally specific attitudes.
Ethics oversight	All experiments were approved by the Research Ethics Committee of the School of Psychological Science at the University of Bristol, UK, under approval code 10874.

Note that full information on the approval of the study protocol must also be provided in the manuscript.

Field-specific reporting

Please select the one below that is the best fit for your research. If you are not sure, read the appropriate sections before making your selection.

☐ Life sciences ☒ Behavioural & social sciences ☐ Ecological, evolutionary & environmental sciences

For a reference copy of the document with all sections, see nature.com/documents/nr-reporting-summary-flat.pdf

Behavioural & social sciences study design

All studies must disclose on these points even when the disclosure is negative.

Study description	The study included three quantitative experiments (each with a secondary qualitative element, allowing participants to explain their quantitative responses).
Research sample	Participants were recruited via online platforms (see 'Recruitment' section, above) to obtain reasonably diverse adult samples quickly and efficiently, in line with current practices in behavioural science research. After exclusions (see 'Data exclusions' section, below), Experiment 1 had 175 participants, aged between 20 and 76 years (M = 41.68, SD = 13.20), Experiment 2 had 275 participants, aged between 19 and 70 years (M = 36.66, SD = 11.53), and Experiment 3 had 223 participants, aged between 19 and 58 years (M = 30.54, SD = 8.71). Our experiments were designed to test general cognitive and social processes that were not expected to depend on specific demographic characteristics.
Sampling strategy	Target sample sizes were determined a priori using G*Power (v3.1) software, as detailed in our preregistrations (available at osf.io/sjw9h). Sampling was conducted via convenience samples of adult participants who chose to take part via online recruitment platforms (see 'Recruitment' section, above), in line with current practices in behavioural science research.
Data collection	All three experiments were hosted online using the Qualtrics survey platform. Participants accessed a link provided by the online recruitment platform (see 'Recruitment' section, above). Responses were recorded by Qualtrics and exported for analysis (see 'Data and code' section, above).
Timing	Data for Experiment 1 were collected on 24-25 November 2022. Data for Experiment 2 were collected on 13-14 April 2023. Data for Experiment 3 were collected on 30 September 2024.
Data exclusions	Exclusion criteria were predetermined, as stated in our preregistrations (available at osf.io/sjw9h). From 222 total responses in Experiment 1, 47 participants were excluded: 18 for failing to complete the survey, and 29 for failing the attention check. From 338 total responses in Experiment 2, 63 participants were excluded: 14 for failing to complete the survey, and 49 for failing the attention check. From 238 total responses in Experiment 3, all participants completed the survey, but 15 participants were excluded for failing the attention check.

Non-participation	After choosing to take part via an online recruitment platform (see 'Recruitment' section, above), no participants declined to give their informed consent. (See 'Data exclusions' above for the numbers of participants who failed to complete the survey.)
Randomization	Participants were randomly allocated to conditions using the 'Randomiser' feature on the Qualtrics survey platform.

Reporting for specific materials, systems and methods

We require information from authors about some types of materials, experimental systems and methods used in many studies. Here, indicate whether each material, system or method listed is relevant to your study. If you are not sure if a list item applies to your research, read the appropriate section before selecting a response.

Materials & experimental systems

n/a	Involved in the study
☒	☐ Antibodies
☒	☐ Eukaryotic cell lines
☒	☐ Palaeontology and archaeology
☒	☐ Animals and other organisms
☒	☐ Clinical data
☒	☐ Dual use research of concern
☒	☐ Plants

Methods

n/a	Involved in the study
☒	☐ ChIP-seq
☒	☐ Flow cytometry
☒	☐ MRI-based neuroimaging

Plants

Seed stocks	*Report on the source of all seed stocks or other plant material used. If applicable, state the seed stock centre and catalogue number. If plant specimens were collected from the field, describe the collection location, date and sampling procedures.*
Novel plant genotypes	*Describe the methods by which all novel plant genotypes were produced. This includes those generated by transgenic approaches, gene editing, chemical/radiation-based mutagenesis and hybridization. For transgenic lines, describe the transformation method, the number of independent lines analyzed and the generation upon which experiments were performed. For gene-edited lines, describe the editor used, the endogenous sequence targeted for editing, the targeting guide RNA sequence (if applicable) and how the editor was applied.*
Authentication	*Describe any authentication procedures for each seed stock used or novel genotype generated. Describe any experiments used to assess the effect of a mutation and, where applicable, how potential secondary effects (e.g. second site T-DNA insertions, mosiacism, off-target gene editing) were examined.*
